# Association between Adherence to Guideline-Recommended Preventive Medications and In-Hospital Mortality among Non-Reperfused ST-Elevation Myocardial Infarction Patients Admitted to a Tertiary Care Academic Center in a Developing Country

**DOI:** 10.5334/gh.394

**Published:** 2020-02-06

**Authors:** Sylvi Irawati, Surya Dharma, Katja Taxis, Thang Nguyen, Nunung Nursyarofah, Bob Wilffert, Eelko Hak

**Affiliations:** 1 University of Groningen, Groningen Research Institute of Pharmacy, PharmacoTherapy, -Epidemiology and -Economics, Groningen, NL; 2 Center for Medicines Information and Pharmaceutical Care, Faculty of Pharmacy, Universitas Surabaya, Surabaya, ID; 3 Department of Clinical and Community Pharmacy, Faculty of Pharmacy, Universitas Surabaya, Surabaya, ID; 4 Department of Cardiology and Vascular Medicine, Faculty of Medicine, University of Indonesia, Indonesian Cardiovascular Research Center, National Cardiovascular Center Harapan Kita, Jakarta, ID; 5 Department of Pharmacology and Clinical Pharmacy, Can Tho University of Medicine and Pharmacy, Can Tho, VN; 6 Indonesian Cardiovascular Research Center, National Cardiovascular Center Harapan Kita, Jakarta, ID; 7 University of Groningen, Department of Clinical Pharmacy & Pharmacology, University Medical Center Groningen, Groningen, NL

**Keywords:** ST-elevation myocardial infarction, STEMI, no reperfusion therapy, guideline adherence, hospital mortality, Indonesia, developing countries

## Abstract

**Background and aims:**

Acute ST-elevation myocardial infarction (STEMI) is a potentially fatal presentation of coronary artery disease (CAD). Evidence of the impact of acute pharmacological interventions in non-reperfused STEMI patients on subsequent events is limited. We aimed to assess the association between adherence to guideline-recommended preventive medications and in-hospital mortality among this high-risk patient population.

**Methods:**

We conducted a cohort study using data obtained from the Jakarta Acute Coronary Syndrome (JAC) Registry database from a tertiary care academic hospital in Indonesia. We included 1132 of 2694 patients with STEMI recorded between 1 January 2014 and 31 December 2016 who did not undergo acute reperfusion therapy. Adherence to guideline-recommended preventive medications was defined as the combined administration of aspirin, clopidogrel, anticoagulants and statins after hospital admission. The main outcome measure was in-hospital mortality.

**Results:**

Overall, 778 of 1132 patients (69%) received the combination of preventive medications. The guideline non-adherent group had significantly more patients with earlier onset of STEMI, higher Killip class and thrombolysis in myocardial infarction (TIMI) score. After adjustments for measured characteristics using logistic regression modeling, exposure to the combination of preventive therapies was associated with a statistically significant lower risk for in-hospital mortality (adjusted odds ratio: 0.46, 95% confidence interval: 0.30–0.70).

**Conclusions:**

Adherence to guideline-recommended preventive medications was associated with lower risk of in-hospital mortality in non-reperfused STEMI patients. The predictors of not receiving these medications need to be confirmed in future research.

## 1. Introduction

Despite a 22% reduction in the median percentage of the age-standardized death rate due to ischemic heart disease (IHD) in the last two decades accross populations worldwide, IHD is still the leading cause of death in the world [1]. IHD was the leading cause of years of life lost (YLL) in a fifth of the developing countries in 2015 [2,3,4,5]. This stresses the magnitude of the burden of IHD as YLL indicates a more appropriate measure of premature death. The YLL calculation considers the time lost associated with IHD death by multiplying the number of deaths at a certain age and the normative standard life expectancy of that age [4,6]. Myocardial infarction (MI) with or without elevation of ST-segment is an acute and serious presentation of IHD [7,8,9,10,11,12]. The risks of death during and within six months after the index date of hospitalization are higher for patients with ST-segment elevation MI (STEMI) than with non-ST-segment elevation MI (NSTEMI) [13].

Acute reperfusion therapy, preferably timely primary percutaneous coronary intervention (pPCI) conducted maximally within 120 minutes since the first medical contact or STEMI diagnosis, is the best management for ischemic STEMI patients transferred from non-capable pPCI hospitals to capable pPCI hospitals [9,11]. Despite continued attempts to implement this emergency care in patients with STEMI presenting at hospitals, up to more than 50% of these patients still did not receive acute reperfusion therapy [14,15], especially in developing countries such as India [15], Indonesia [15,16,17,18,19], Phillipines [15], and Vietnam [20]. Even in developed countries, around 20%–30% of patients with STEMI who were eligible for reperfusion still failed to receive this evidence-based intervention [21,22,23].

In developing countries like Indonesia, Malaysia, and the Phillipines, late presentation at hospitals (defined as arrival >12 h from symptom onset) contributes to the low rate of pPCI utilisation. A targeted shorter time between the symptom onset and calling an ambulance or presenting at pPCI-capable hospitals was unachieved due to long distance to reach pPCI-capable centers and geographical barriers [15]. In Jakarta, traffic overcrowd, patient-related factors, pre-hospital diagnosis and treatment delay, lack of collaboration between hospital and physicians, and lack of ambulance organisation might cause the late presentation [16].

Inconsistencies exist in the evidence on the benefit of acute reperfusion therapy, primarily pPCI, over pharmacological therapy alone in patients with STEMI who present late [24,25,26]. International guidelines have not directly specified management on this type of STEMI patients [9,11,27]. It is suggested the same classes of medications such as dual antiplatelet therapy (DAPT), anticoagulants, and statins applied for reperfused patients are also applied for non-reperfused patients as one package [25]. However, this suggestion is based on trials in STEMI patients whom more than 50% of them were also reperfused by fibrinolytic agents [28] and in the era where other preventive medications, especially DAPT and statins, were not routinely used or not reported [27,28,29,30]. More over, populations understudied in these trials are at lower risk of in-hospital mortality compared to a ‘real-world’ setting. More than 70% of these populations presented with Killip class = 1 [28,30] or without heart failure [29].

The effect size of using all classes of these preventive medications on in-hospital mortality in non-reperfused STEMI patients, especially in real-world developing countries where the risk is higher, is unclear. The objective of this study was to assess the association between adherence in presribing a combination of guideline-recommended preventive medications and in-hospital mortality among non-reperfused STEMI patients admitted to a tertiary care academic hospital in Indonesia, a developing country.

## 2. Methods

### 2.1. Study design and setting

We performed a cohort study among non-reperfused STEMI patients prospectively registered in the Jakarta Acute Coronary Syndrome (JAC) Registry database from 1 January 2014 to 31 December 2016. The registry was set up in 2007 and is prospectively collecting and managing data on characteristics, management and outcomes of patients with ACS admitted to the emergency department (ED) of the National Cardiovascular Center Harapan Kita (NCCHK) which is the largest tertiary cardiac referral hospital in Jakarta, the capital of Indonesia. The hospital is JCI (Joint Committee International) accredited.

Data were collected prospectively and consecutively using a standardized registry form. In addition, all data were recorded in the database of the JAC registry after the patient was discharged. Data quality was maintained and verified regularly by the primary investigator of the registry (SD). The hospital provides a 24/7 cardiovascular service, including primary percutaneous coronary intervention (pPCI) and is hosting the regional STEMI network [17,18,19]. More details on the STEMI network characteristics in Jakarta was previously described [17]. In brief, patients with acute STEMI will be transferred to the nearest PCI center for primary PCI. An electrocardiogram (ECG) transmission system is adopted to confirm the STEMI diagnosis. Since the hospital provide a 24/7 service, most of the STEMI cases were transferred to the national cardiovascular hospital, particularly during off-hours. A pre-hospital triage form was developed to improve the acute phase management [19].

The reporting of this study was in accordance with the Strengthening the Reporting of Observational Studies in Epidemiology (STROBE) guideline [31]. This study has been approved by the local Institutional Review Board (IRB) (No. LB.02.01/VII/233?KEP.004/2018).

### 2.2. Patients and exposure

A total number of 2694 patiens with STEMI were recorded. Of these, 1132 patients (42%) did not receive acute reperfusion therapy and were included in the final analysis. The majority (74.9%) of the non-reperfused patients were late presenters, defined as presenting >12 h from symptoms of STEMI [25]. Diagnosis of STEMI was made based on the presence of typical chest pain and ST-segment elevation (≥0.1 mV) in two or more contiguous leads on the initial ECG [16,17,19]. Acute reperfusion therapy was defined as pPCI or fibrinolytic therapy. Of 1562 patients who received acute reperfusion therapy, 91% were treated with pPCI.

Patients were divided into two groups: guideline-adherent group and guideline non-adherent group. We used the American College Cardiology (ACC)/American Heart Association (AHA) and European Society of Cardiology (ESC) guidelines for the management of patients with STEMI as these were adopted by the Indonesian guideline [9,32,33]. Adherence to guideline-recommended medications for each patient was defined as receiving a combination of all four medications (aspirin, clopidogrel, parenteral anticoagulants and statins) after hospital admission. Clinicians assured that all patients received their medications during the hospital stay. Patients who received the combination of all four medications were included into the guideline-adherent group. Patients who received less than four of these medications, i.e. 0–3 of the recommended medications were included into the guideline non-adherent group.

### 2.3. Outcomes, follow-up and potential confounders

The primary outcome was in-hospital mortality as recorded in the registry. Patients were followed-up from the date of admission to the date of death in hospital, discharge from the hospital, or the end of the study period. Potential confounders were patient’s characteristics considered to be associated with the use of at least one type of the in-hospital preventive medications or in-hospital mortality found in previous studies in patients with acute MI and ACS [20,27,28,29,30,34,35]. Only one study, observing patients with ACS, reported a clear association between several patient’s characteristics (type of MI, cardiac enzyme, and Killip class) and the optimal use of these medications [35].

Based on the completeness of variables observed in the registry (missing observation less than 5%), the following variables were selected as potential confounders in our study: age (categorized into ≤65 or >65 years), sex, family history of coronary artery disease (CAD), hypertension, diabetes, dyslipidemia, smoking status, Killip class (1 or >1), onset of STEMI (≤12 or >12 h), anterior MI, Thrombolysis in Myocardial Infarction (TIMI) score (<4 or ≥4), and other cardiovascular-preventive medications administered after hospital admission.

### 2.4. Statistical analysis

The data on categorical variables were presented as proportions. All comparisons in variable distributions between groups were tested using c^2^-test for each variable. A binary logistic regression model was used to estimate the crude association between exposure status and in-hospital mortality, and was presented as an odds ratio (OR) with 95% confidence interval (CI). We applied multivariable logistic regression analysis to obtain an adjusted OR by including significant potential confounders from the univariable analysis. All statistical analysis to test the difference of variable distribution between groups were two-sided and *p* < 0.05 were considered significant. All analyses were performed using the Statistical Package for the Social Sciences, V21 (SPSS 21).

## 3. Results

Out of total 1132 non-reperfused STEMI patients, the majority (81.4%) were middle-aged (<65 years), males (85.1%) and referred from other hospitals (52.9%). The three most common IHD risk factors identified were smoking (62.8%), hypertension (51.3%) and diabetes (32.2%). The majority of patients had an anterior MI (61.7%).

**Figure 1:**
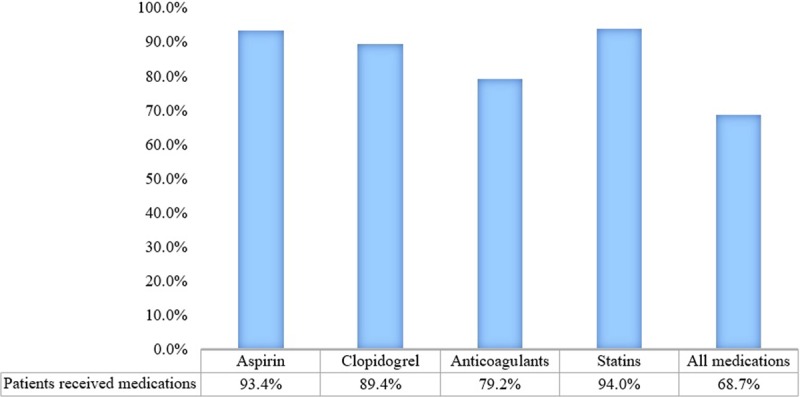
The level of adherence in prescribing individual medications (aspirin, clopidogrel, anticoagulants, statins) and all-combined medications in non-reperfused STEMI patients during hospitalization.

There were 778 patients (68.7%) in the guideline-adherent group and 354 patients (31.3%) in the guideline non-adherent group (Figure 1). There were more patients with family history of CAD, hypertension, and smoking habit in the guideline-adherent group compared to those in the guideline non-adherent group, although the differences were not statistically significant.

Three key significant variables correlated with non-adherence were all indicators of a more severe/acute STEMI: higher TIMI score (≥4), higher Killip class (>1), and more recent onset of STEMI (≤12h). The guideline non-adherent group were also less likely to receive angiotensin-converting enzyme inhibitors/angiotensin receptor blockers (ACEIs/ARBs) within 24 h after hospitalization compared to the guideline adherent group (Table 1).

**Table 1:** Baseline characteristics of patients with ST-segment elevation myocardial infarction not receiving acute reperfusion therapy.

Patient’s characteristics^a^	Guideline-adherent (N = 778)	Guideline non-adherent (N = 354)	*p*-value^b^	Missing observation (% of total N)

Age > 65 years, n (%)	144 (18.5)	67 (18.9)	0.867	0
Males, n (%)	669 (86.0)	294 (83.1)	0.198	0
Source of referral, n (%)				
Inter-hospital	399 (51.2)	200 (56.5)	0.437	0
Walk in/ambulance	335 (43.1)	137 (38.7)		
Intra-hospital	30 (3.9)	12 (3.4)		
Primary physician	14 (1.8)	5 (1.4)		
IHD risk factors, n (%)				
Family history of CAD	134 (17.2)	48 (13.6)	0.120	0
Hypertension	406 (52.2)	175 (49.4)	0.391	0
Diabetes	250 (32.1)	114 (33.2)	0.981	0
Dyslipidemia	227 (29.2)	99 (28.0)	0.677	0
Smokers	499 (64.1)	212 (59.9)	0.170	0
Hospital findings, n (%)				
Onset of STEMI ≤ 12 h	161 (20.8)	113 (32.4)	**<0.0005**	10 (0.9)
Anterior MI	480 (61.9)	219 (62.4)	0.863	5 (0.4)
Killip class > 1	270 (35.1)	158 (46.7)	**<0.0005**	25 (2.2)
TIMI score ≥ 4	367 (48.3)	177 (55.8)	**0.024**	55 (4.9)
Random blood glucose ≥ 200 mg/dL	198 (25.8)	97 (29.4)	0.220	35 (3.1)
Other medications administered after admission, n (%)				
GP IIb/IIIa inhibitors	11 (1.4)	5 (1.4)	0.998	0
Beta blockers	214 (27.5)	96 (27.3)	0.935	2 (0.2)
ACEIs/ARBs	421 (54.1)	167 (47.4)	**0.038**	2 (0.2)

^a^ Percentage was calculated as the number of patients with a certain characteristic per total number in each group excluding any missing observations. ^b^ χ^2^ test, bold type indicated statistical significance. ACEIs = angiotensin-converting enzyme inhibitors, ARBs = angiotensin receptor blockers, CAD = coronary artery disease, GP = glycoprotein, h = hour, IHD = ischemic heart disease, MI = myocardial infarction, STEMI = ST-segment elevation myocardial infarction, TIMI = thrombolysis in myocardial infarction.

All included patients had a complete follow-up. Overall, in-hospital mortality of non-reperfused STEMI patients within the period of observation was 12.2%. As expected, the non-surviving group had significantly more patients with Killip class >1 and TIMI score ≥4 and less patients treated with ACEIs/ARBs within 24 h (Table 2).

**Table 2:** Distribution of potential confounders between non-reperfused STEMI patients who were alive and deceased during hospitalization.

Potential confounders^a^	Deceased (N = 138)	Alive (N = 994)	*p*-value^b^	Missing observation (% of total N)

Hospital findings, n (%)				
Onset of STEMI ≤ 12 h	46 (34.1)	228 (23.1)	**0.005**	10 (0.9)
Killip class > 1	95 (72.5)	333 (34.1)	**<0.0005**	25 (2.2)
TIMI score ≥ 4	98 (81.7)	446 (46.6)	**<0.0005**	55 (4.9)
Medications administered after admission, n (%)				
ACEIs/ARBs	39 (28.5)	549 (55.3)	**<0.0005**	2 (0.2)

^a^ Percentage was calculated as the number of patients with a certain characteristic per total number in each group excluding missing observation. ^b^ χ^2^ test, bold type indicated statistical significance. ACEIs = angiotensin-converting enzyme inhibitors, ARBs = angiotensin receptor blockers, h = hour, STEMI = ST-segment elevation myocardial infarction, TIMI = thrombolysis in myocardial infarction.

In univariable analysis, guideline-adherence in non-reperfused STEMI patients was associated with lower odds of in-hospital mortality (OR: 0.32, 95% CI 0.22–0.46) (Table 3). After adjustment for potential counfounders (onset of STEMI, Killip class, TIMI score, and administration of ACEIs/ARBs within 24 h after hospitalization), the adjusted OR was 0.46 (95% CI 0.30–0.70). When the association between guideline-adherence and in-hospital mortality was analysed in multivariable analysis that also adjusted for other potential confounders (age, sex, family history of CAD, hypertension, dyslipidaemia, diabetes, smoking status, anterior MI, onset of STEMI, Killip class, TIMI score, and ACEIs/ARBs administration), the result did not change (adjusted OR: 0.46, 95% CI 0.30–0.70).

**Table 3:** Association between adherence to guideline-recommended medications and in-hospital mortality in non-reperfused STEMI patients.

	In-hospital mortality		Adjusted OR (95% CI)^a^

	N of death/total (%)	Crude OR (95% CI)	

Guideline-adherent^b^	62/778 (8.0)	0.32 (0.22–0.46)	0.46 (0.30–0.70)
Guideline non-adherent^c^	76/354 (21.5)		

^a^ Adjusted for onset of STEMI, Killip class, TIMI score and administration of ACEIs/ARBs. ^b^ Guideline-adherent was defined as receiving a combination of all four medications (aspirin, clopidogrel, anticoagulant and statins). ^c^ Guideline non-adherent was defined as receiving less than four medications. ACEIs = angiotensin-converting enzyme inhibitors, ARBs = angiotensin receptor blockers, CI = confidence interval, OR = odds ratio, STEMI = ST-segment elevation myocardial infarction, TIMI = thrombolysis in myocardial infarction.

## 4. Discussion

Almost 70% of non-reperfused STEMI patients received all four medications (DAPT [aspirin and clopidogrel], anticoagulants, and statins) as recommended by guidelines. This level of adherence was significantly associated with a 54% reduction in the odds of in-hospital mortality after adjusting for potential confounders. It seems that the acuteness and severity of patients admitted with STEMI correlated positively with guideline non-adherence.

### 4.1. Comparison with other studies

Most previous studies observed guideline adherence of preventive medications administered at-discharge [36,37]. Two slightly similar studies looked at a different set of medications in patients with ACS in Vietnam [20] and India [35]. In Vietnam, guideline adherence was defined as prescribing DAPT, beta blockers, ACEIs/ARBs, and statins both within 24 h after hospital admission and at-discharge [20], while in India, it was defined as receiving DAPT, heparin, beta blockers, and statins in hospital only [35]. The proportion of STEMI patients in both studies was under 40% with unclear report on the number of non-reperfused patients. However, pPCI was only received by 25% and 12% of ACS patients in both studies respectively, suggesting substantial patients with STEMI were not reperfused. The level of guideline-adherence in both studies were lower than our study, 46% in Vietnam and 40% in India [20,35]. This difference is probably due to the difference of population understudied and the definition of guideline adherence. Due to incompleteness of some clinical data, we observed less types of medication to avoid misclassification of adherence.

The trend of higher risk patients (indicated by a higher Killip class and TIMI score) not receiving guideline-recommended preventive medications is comparable with the Indian study. Killip class >1 and positive cardiac enzyme significantly predicted sub-optimal in-hospital preventive medications in Indian ACS patients [35]. A phenomenon of higher risk patients less likely receiving guideline-recommended treatment compared to lower risk patients, known as a treatment-risk paradox, has been investigated in patients with NSTEMI [36,38,39]. The causes of this paradox have not been adequately studied. The tendency of physicians to avoid invasive intervention in patients perceived to be at higher risk of procedure-related complications, such as bleeding or stroke, has been suggested to partly cause this paradox in NSTEMI patients [38]. A study on predictors of not receiving secondary preventive medications at-discharge in patients with STEMI found chronic oral anticoagulation and in-hospital bleeding as two important predictors of not receiving aspirin or clopidogrel [40]. Since the level of adherence in the use of anticoagulants alone was the lowest compared to the others in our study, thus contributes to the overal level of adherence, we suggested some conditions that would increase a perceived potential adverse effect or complication might discourage the administration of triple antithrombotic medications in higher risk or sicker patients, e.g. higher risk of bleeding, oral anticoagulant co-medications. We also can not rule out patient’s factors such as refusal of receiving medications due to personal reasons.

Moreover, the management of STEMI is not guided by a risk stratification as in NSTEMI [32,33]. It is assumed that similar treatment will be beneficial for all patients with STEMI, even more in the non-reperfused ones. However, evidence used to support this suggestion is based on patients with lower risk of in-hospital mortality compared to a ‘real-world’ setting [28,29,30]. Thus, there is still a gap of knowledge on the benefit of secondary preventive medications in this subpopulation, especially when the perceived potential adverse effects might counterbalance the benefits in higher risk patients and when there is no clear stratification of risk in this population.

While optimal use of in-hospital medications showed a non-statistical significant trend towards lower in-hospital mortality in Indian patients with ACS [35], our study found otherwise. Different types of medications being assessed and population understudied likely contribute to different results. A recent observational study investigated an association between UFH or enoxaparin and in-hospital mortality in non-reperfused STEMI patients who the majority also received aspirin (95%) and clopidogrel (71%). Information on the use of other preventive medications including statins was not reported. In the subgroup analysis, the use of UFH was associated with a lower risk of the odds of in-hospital mortality in clopidogrel users only (OR: 0.62, 95% CI 0.41–0.94) while this effect was not significant in clopidogrel non-users. The higher protective effect of a combined medication shown in our study suggests that an early use of statins (within 24-h after hospital admission) might contribute to further reduction in in-hospital mortality. Nevertheless, little is known on pharmacological mechanism of statins in concert with DAPT and anticoagulants in late presenter non-reperfused STEMI patients who has a higher prothrombotic state as a specific pathophysiological condition [27].

### 4.2. Limitations

Several limitations should be acknowledged when interpreting the result of this study. First, since we only measured the level of adherence to the medications at hospital admission (mostly within 24 h) and did not directly observe patients taking the medications during hospitalization, this may have led to some misclassification of exposure. However, as the hospital is JCI accredited, this process was monitored daily by nurses and pharmacists and therefore should not have resulted in a large bias.

Secondly, since the OR is a statistic output of the logistic regression and the outcome rate was not rare (>10% in non-exposed group) the OR most likely will overestimate the true relative risk (RR). After correction of the adjusted OR using formula developed by Zhang and Yu [41,42], the adjusted RR was 0.52 (95% CI 0.35–0.75) which is still highly significant.

Thirdly, we could not assess conditions that contraindicated the use of DAPT, anticoagulants, and statins, such as history of intolerance, active gastrointestinal bleeding, or active liver disease [20,43,44,45,46]. The prevalence of conditions that contraindicated the use of these medications in STEMI patients has also rarely been reported in previous studies. In one study, all patients with STEMI were eligible for receiving aspirin/clopidogrel and only 0.9% were not eligible for receiving statins [43]. Consequently, although we cannot exactly know the effect of contra-indications on in-hospital mortality, this might overestimate the level of guideline adherence.

Finally, although the association between guideline-adherence and in-hospital mortality has been adjusted for potential confounders, the possibility of confounding by other unmeasured potential confounders, such as prior CVD (MI, stroke), medications or treatment used prior to hospital admission, lipid profile, baseline systolic blood pressure (SBP), left ventricular ejection fraction (LVEF) profile, body mass index (BMI), and other comorbidities (asthma/chronic obstructive pulmonary disease [COPD]; renal dysfunction; heart failure) or healthy lifestyle, may have biased our finding away from the null.

### 4.3. Future directions

Our findings should reinforce the use of guideline recommended secondary preventive medications in non-reperfused STEMI patients. The indication of treatment-risk paradox that may challenge the adherence to guideline recommended preventive medications in this subpopulation is intriguing. Several strategies to minimize this paradox, such as reimbursement (pay for performance incentives), modifying the practice setting (e.g. practice aids), and multidisciplinary approach, has been proposed in the management of NSTEMI patients [39]. However, due to the difference in management between STEMI and NSTEMI, the causes of this paradox may not be the same. The presence of this paradox needs to be confirmed to identify a more appropriate strategy. The predictors of not receiving medications in this subpopulation need to be identified in future research.

## 5. Conclusions

Approximately two-thirds of non-reperfused STEMI patients admitted to a tertiary academic hospital in Indonesia, a developing country, received in-hospital preventive medications according to international guidelines. This level of adherence has contributed to reduction of the odds of in-hospital mortality by 54%. The predictors of not receiving these preventive medications need to be further investigated.
